# Fas-positive lymphocytes are associated with systemic inflammation in obstructive sleep apnea syndrome

**DOI:** 10.1007/s11325-018-1713-8

**Published:** 2018-08-31

**Authors:** Joanna Domagała-Kulawik, Iwona Kwiecień, Piotr Bielicki, Tomasz Skirecki

**Affiliations:** 1 Department of Internal Medicine, Pulmonary Diseases and Allergy, Medical University of Warsaw, ul. Banacha 1a, 02 097 Warsaw, Poland; 2 Laboratory of Flow Cytometry, Department of Internal Medicine and Hematology, Military Medical Institute, ul. Szaserow 128, 04 141 Warsaw, Poland; 3 Laboratory of Flow Cytometry, Department of Anesthesiology and Intensive Care, Centre of Postgraduate Medical Education, ul. Marymoncka 99/103, 01 813 Warsaw, Poland

**Keywords:** OSAS, Lymphocytes, Fas receptor, Systemic inflammation

## Abstract

**Purpose:**

Obstructive sleep apnea syndrome (OSAS) is associated with alterations in immune system which may lead to serious complications. The aim of this study was to explore lymphocyte populations in OSAS with special attention to the Fas-positive cells.

**Methods:**

Fifty-one patients with confirmed OSA and 20 healthy subjects were investigated. The OSA severity indices, data concerning comorbidities, and markers of inflammation and metabolic disorders were collected. Flow cytometry was used to analyze the lymphocyte profile and expression of Fas receptors (CD95). Concentration of adiponectin, IL-1β, TNF-α, and sFas were measured.

**Results:**

Proportions of Fas-positive cells in the pool of CD4+ and Fas-positive in the pool of CD8+ cells in the blood of patients were significantly increased when compared with healthy subjects (74.5% vs. 65.6% and 78.8% vs.70.9%, respectively, *p* < 0.05). No correlation with OSA severity was found. However, the proportion and number of Fas+ cells were elevated in obese patients, in non-smokers, and in patients suffering from COPD and hypertension. There were several significant relations of Fas+ cells with inflammatory markers of systemic inflammation.

**Conclusion:**

Lymphocytes with the expression of Fas receptor are associated with systemic inflammation in OSAS.

## Introduction

Obstructive sleep apnea syndrome (OSAS) is a breathing disorder during sleep, which is known to be connected with obesity, metabolic syndrome, and the risk of cardiovascular complications and especially risk of arterial hypertension. OSAS and related complications are accompanied by systemic inflammation [1, 2]. We previously reported significant changes in the proportion of blood lymphocytes in OSA patients. The proportion of B cells, Th/Tc ratio, and adiponectin concentration were lower but, the proportion of Tc, NK, NKT-like, and HLA-DR positive T cells were elevated in OSAS patients when compared with healthy subjects and these changes correlated with metabolic complications [3]. In this study, we aimed to further evaluate blood lymphocyte characteristics by the analysis of the expression of Fas receptor on the main lymphocyte subtypes. Fas (CD95) is a death receptor and belongs to the tumor necrosis factor receptor (TNFR) superfamily. Recently, its role in the modulation of immune response was described and it was found that the role of Fas/FasL pathway is wider than previously thought [4–6]. It was described, among others, that Fas/FasL signaling contributes to antigen-presenting cell activation, activation-induced cell death of T cells and thus to a maintenance of T cells homeostasis, Th17 differentiation, and precocious differentiation of memory T cells [4–6]. Therefore, the relationship of Fas-positive cells with markers of systemic inflammation, metabolic complications, OSA severity, and inflammatory cytokine concentration was of interest to this study.

## Material and methods

**Fig. 1:**
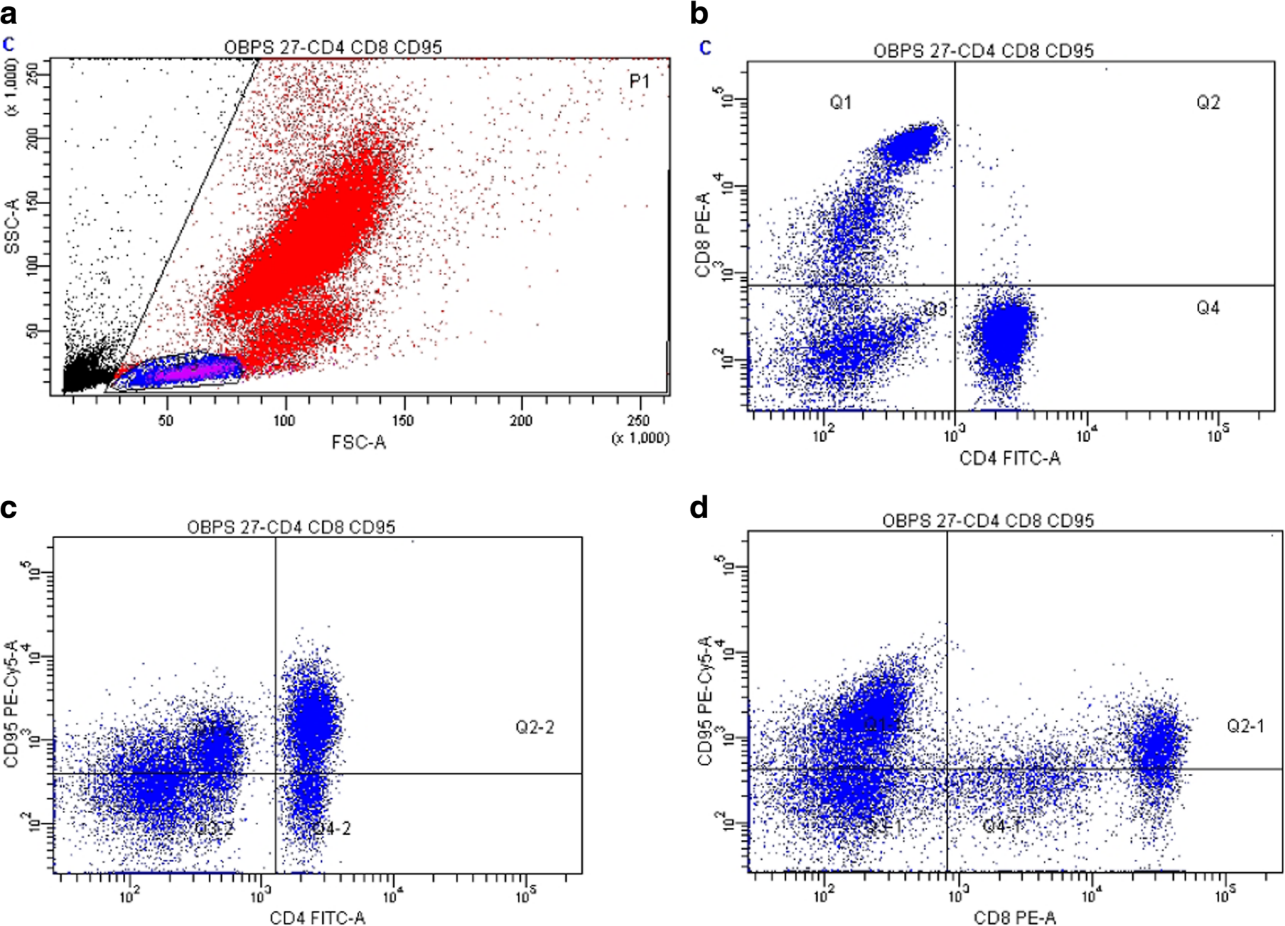
Representative flow cytometry analysis of Fas expression in blood lymphocytes in OSA patient. **a** Morphological gate for lymphocytes is shown, **b** then CD4+ and CD8+ cells are gated. Analysis of Fas (CD95) expression on **c** CD4+ and **d** CD8+ T cells

Fifty-one patients with confirmed OSAS were enrolled into the study. The diagnosis of OSAS was established in accordance with the American Academy of Sleep Medicine (AASM) and Polish Respiratory Society recommendations [7, 8]. A polysomnography test (PSG) was executed using the Alice 4 apparatus (RESPIRONICS, USA). The criteria of diagnosis were the apnea/hypopnea index (AHI) value over 5 and the Epworth Sleepiness Scale (ESS) over 10 points. The control group consisted of 20 healthy volunteers without any chronic disease. The study was approved by the Ethics Committee of the Medical University of Warsaw and all the participants gave informed consent. The venous blood samples were collected before breakfast, early in the morning. All the analyses were performed right after blood collection. We analyzed the proportions of the following lymphocyte subtypes: T cells, B cells, T helper (Th) and T cytotoxic cells (Tc), natural killer (NK), natural killer T cells (NKT-like), and T cells with HLA-DR expression by Simultest (BD, San Jose, California, US). Flow cytometry analysis was performed as previously described [3]. For the analysis of Fas receptor expression on T cells, the following cocktail of antibodies was used: CD4-FITC/CD8-PE/CD95-PE-Cy5. Briefly, anti-CD45-FITC and anti-CD14-PE were used for the lymphocyte gate setting at FSC/SSC graph (Fig. 1). Negative isotype controls with IgG1-FITC/IgG2a-PE were applied. The analyses were performed using FACS Canto II flow cytometer (Becton-Dickinson, San Jose, California) and Diva software (BD). Ten thousand lymphocytes were collected. Geometric mean fluorescence (GMF) intensity of Fas staining on T cells was measured. The serum concentrations of adiponectin, soluble Fas, interleukin-1β (IL-1β), and tumor necrosis factor (TNF-α) were measured using commercially available ELISA kits: Human Total Adiponectin/Acrp30 Immunoassay kit, HS ELISA Human Fas/TNFRSF6, Human IL-1 beta/IL-1F2, Human TNF-alpha Quantikine ELISA Kit (R&D System, USA), according to the prescription by the producer.

### Statistical analysis

For data comparison, the Mann-Whitney *U* test was applied. A *p* value of less than 0.05 was regarded as significant. The relationships between the data were examined by the Spearman’s rank correlation coefficient. Correlations with both *r* ≥ 0.3 and *p* < 0.05 were considered relevant.

## Results

Of 51 patients with OSAS (35 men, 69%), mean age was 58 years (range 36–82); 33% were smokers with mean 15 pack-years. The control group of 20 subjects (8 men, 40%) had a mean age of 46 years (range 30–76) and normal BMI; 50% were smokers with mean 32 pack-years. For these characteristics, the control group did not differ significantly from the study group. The following OSAS indices characterized the patient group: mean BMI was 31.7 kg/m^2^ and obesity was recognized in 52%; mean AHI was 53 ± 21, ODI was 31 ± 19, EES was 12.2 ± 5.3, and mean lowest peripheral capillary oxygen saturation (SpO_2_) was 76 ± 9%. Severe OSAS was categorized in 86% of patients. The mean values of white blood cell count, concentration of hemoglobin, plasma glucose, total cholesterol (TC), triglycerides (TG), high-density lipoprotein cholesterol (HDL-C), and C reactive protein (CRP) were within normal range. However, in 11% of the patients, the concentration of TG was higher than 200 mg/dL and in 49%, cholesterol > 200 mg/dL. In 52% of patients, features of metabolic syndrome were recognized. Distribution of patients in each group of OSAS complication score (defined according to [3]) was as follows: score 0 (4), score 1 (14), score 2 (19), score 3 (7), score 4 (4), and score 5 (1). Hypertension was recognized in 72% and COPD in 7% of patients.

The gating strategy for the Fas expression analysis is shown in Fig. 1. We present the proportion of Fas-positive cells as a percentage of all lymphocytes (Fas+CD4+ and Fas+CD8+ cells) as well as percentage of Fas-positive cells among all CD4+ cells (%Fas in CD4+) and among all CD8+ cells (%Fas in CD8+). As for the total cell counts, there were mean 464 ± 242 Fas+CD4+ cells/μL, and 494.4 ± 272 Fas+CD8+ cells/μL of peripheral blood. We found significantly higher proportions of Fas-positive cells in the OSAS patients than in the control subjects (Table 1). In the OSAS patients, the expression of Fas (expressed as GMF) on CD4+ cells was 766.6 ± 312 and it significantly correlated with the Fas expression on CD8+ cells (*r* = 0.7, *p* < 0.05) which was 693.4 ± 271. The age and sex had no influence on Fas+ cells proportion (apart from weak correlation of proportion of Fas+CD8+ cells with age).

**Table 1:** Proportion of T lymphocytes with expression of Fas in the blood of OSA patients and control group and the relationship with clinical data and inflammatory markers. Data expressed as median values and quartiles p25–p75, *p* < 0.05, and *r* ≥ 0.3 are considered significant

		Fas^+^CD4^+^		Fas^+^CD8^+^	
		Fas^+^CD4^+^ [% of lymphocytes]	Fas^+^ [% of all CD4^+^cells]	Fas^+^CD8^+^ [% of lymphocytes]	Fas^+^ [% of all CD 8^+^cells]
	Patients	25.9	74.5	28.5	78.8
		20.4–33.5	58.7–93.7	22.0–40.1	65.7–92.8
	Control group	28.1	65.6	19.0	70.9
		23.6–31.4	60.2–72.6	16.6–22.7	58.8–76.0
	*p*	> 0.05	0.05	0.002	0.004
Relation with:					
1. Other immune cells and mediators of inflammation	PMN	Fas^+^CD4^+^/μL *r* = − 0.3, *p* < 0.05		Fas^+^CD8^+^/μL *r* = − 0.3, *p* < 0.05	
	PLT	GMF Fas^+^CD4^+^*r =* −*0.3, p < 0.05*		Fas^+^CD8^+^ % *r* = − 0.3, *p* < 0.05 GMF Fas^+^CD8^+^*r* = − 0.3, *p* < 0.05	
	CD19%			Fas^+^CD8^+^ % *r* = − 0.3, *p* < 0.05	
	CD19/ul	Fas^+^ CD4^+^ % *r* = − 0.3, *p* < 0.05		Fas^+^CD8^+^/μL *r* = − 0.4, *p* < 0.05	
		GMF Fas^+^ CD4^+^*r* = − 0.4, *p* < 0.05			
	NK%	Fas^+^ CD4^+^ % *r* = 0.3, *p* < 0.05			
	NK/ul	Fas^+^CD4^+^/μL *r* = 0.5, *p* < 0.05		Fas^+^CD8^+^/μL *r* = − 0.3, *p* < 0.05	
	NKT%			Fas^+^CD8^+^ % *r* = 0.5, *p* < 0.05	
	CD3+/HLA-DR + %	Fas^+^CD4^+^ % *r* = 0.3, *p* < 0.05		Fas^+^CD8^+^ % *r* = 0.4, *p* < 0.05	
	CD3+/HLA-DR+/ul	Fas^+^CD4^+^/μL *r* = 0.4, *p* < 0.05		Fas^+^CD8^+^/μL *r* = 0.6, *p* < 0.05	
	Adiponectin/BMI	Fas^+^CD4^+^/μL *r* = 0.4, *p* < 0.05			
		sFas concentration *r =* − 0.3, *p* < 0.05			
2. OSA indices	EES	Fas^+^CD4^+^ % *r* = − 0.4, *p* < 0.05		Fas^+^CD8^+^ % *r* = 0.06, *p* > 0.05	
	SpO_2_	GMF Fas^+^CD4^+^*r* = − 0.3, *p* < 0.05			
3. Metabolic disorders	BMI	Fas^+^CD4^+^ % *r* = 0.3, *p* < 0.05		Fas^+^CD8^+^ % *r* = 0.3, *p* < 0.05	
	TG	Fas^+^CD4^+^/μL *r* = 0.4, *p* < 0.05			
4. Comorbidities	Non-significant elevation of Fas+CD4 and Fas+CD8 cells in hypertension and COPD				
5. Smoking history	Pack-years smoked	Fas^+^CD4^+^ % *r =* − 0.3, *p* < 0.05		Fas^+^CD8^+^ % *r =* − 0.4, *p* < 0.05	
		GMF Fas^+^CD4^+^*r =* − 0.5, *p* < 0.05		GMF Fas^+^CD8^+^*r =* − 0.3, *p* < 0.05	

*BMI* body mass index

*COPD* chronic obstructive pulmonary disease

*ESS* Epworth Sleepiness Scale

*GMF* geometric mean fluorescence intensity

*PLT* platelets

*PMN* polymorphonuclear cells

*sFas* soluble Fas

*SpO*_*2*_ peripheral capillary oxygen saturation

*TG* triglycerides

The median concentration of sFas was 1911.4 (1759.4–2053.4) pg/mL and did not differ when compared with that of the control group (2062.7 (1826.1–2090.1) pg/mL). No correlations of sFas concentration with clinical data, OSA severity, nor cell proportions were found. We did not find any correlations of IL-1 (0.3 (0.1–05) pg/mL) nor TNF-α (1.22 (0.7–13.9) pg/mL) concentration with patient data.

**Fig. 2:**
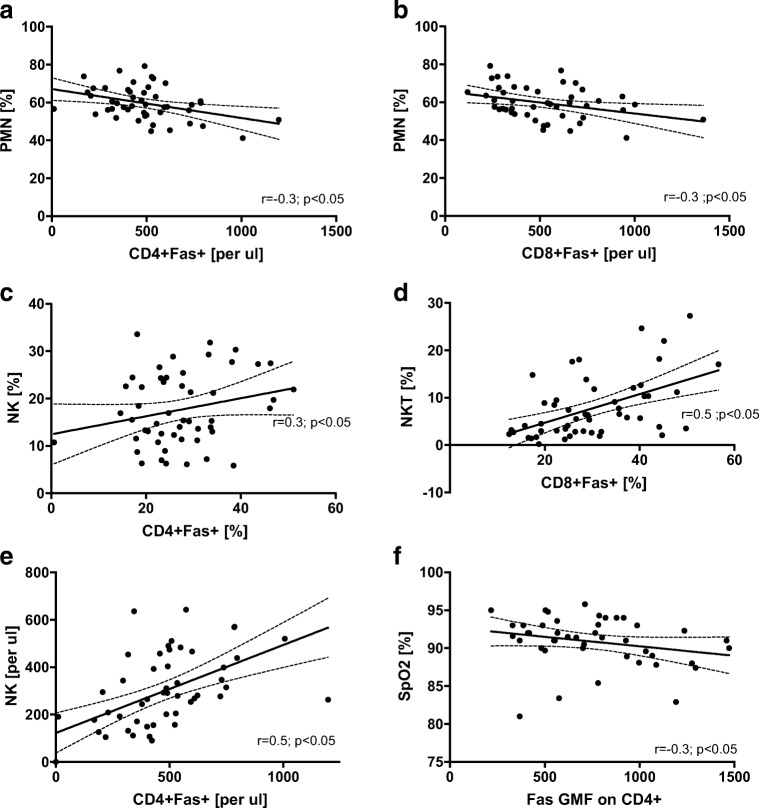
Scatter plots of correlations between Fas-positive T cells and other variables. **a** Count of CD4+Fas+ lymphocytes and polymorphonuclear cells (PMN) percentage. **b** Count of CD8+Fas+ lymphocytes and PMN percentage. **c** Proportion of CD4+Fas+ cell and NK cell percentage. **d** Proportion of CD8+Fas+ lymphocytes and NKT cell percentage. **e** Counts of CD4+Fas+ cells and NK cells. **f** Geometric mean fluorescence (GMF) of Fas receptor staining on CD4+ cells and oxygen saturation (SpO_2_). Dashed lines show 95% confidence intervals

We analyzed the relationships between Fas-positive T cell percentage with (a) other immune cells and mediators, (b) OSA indices, (c) metabolic disorders, (d) comorbidities, and (e) smoking history. The results of this analysis are presented in Table 1 and Fig. 2.

## Discussion

There is a growing body of evidence that the nature of OSAS and systemic complications of this pathology are connected with immune system alterations. However, precise data on the nature of inflammation are still scanty. In our previous study, we observed significant changes in the populations of circulating inflammatory cells, i.e., lymphocytes B, cytotoxic T (Tc), NK, and NKT-like cells, and in the concentration of adiponectin in OSAS. Our recent investigation revealed an involvement of death receptor Fas in the inflammatory process of severe OSA. Here, we presented Fas-positive cells as proportion of lymphocytes, as proportion of lymphocyte subpopulations, as absolute number, and as cell surface expression of this receptor. It allowed us to assess objectively the expression of the Fas receptors among T cells. Our study group consisted of patients with severe OSAS and our findings might reflect adaptive processes of the immune system. The negative correlation of oxygen saturation with Fas expression seems to confirm this hypothesis. Recently obtained data showing the pivotal role of the Fas/FasL signaling pathway in maintaining the homeostasis of immune system [6] supports the value of our study.

Our current findings on the Fas expression are especially marked in the CD8+ cell population. Dyugovskaya et al. showed that CD8+ cells form the most affected population in OSAS [9]. Tan et al. found a high proportion of CD8+ cells and a low proportion of CD4+ cells according to the AHI in children suffering from OSAS [10]. CD8+ cells constitute the T cell subpopulation which contributes to the formation of atherosclerotic lesions [11]. We previously reported an elevated proportion of lymphocytes with Fas expression in patients with lung cancer and in COPD [12, 13]. In the current study, the proportion of Fas-positive cells was much higher in OSA/COPD overlap syndrome which is in line with the hypothesized role of CD8+ cells in COPD pathogenesis. In our previous studies, tobacco smoking was investigated as a factor capable of affecting the immune system. In fact, the proportion of Fas-positive lymphocytes was highly correlated with the intensity of tobacco exposure [12, 13]. Unexpectedly, we found an opposite result in the present study. However, as we found previously, the influence of smoking was striking when smoking history exceeded 20 pack-years [our unpublished data]. Of note, in this current study subjects with short exposure to tobacco smoke were included.

We carefully analyzed the relationship of Fas-positive cell populations with clinical and laboratory parameters. Interestingly, we did not observe any relation of Fas receptor expression with OSAS indices apart from SpO_2_. As in our previous report, a much stronger influence of metabolic and cardiovascular complications on immune cells was observed in this study. This relationship has also been reported by many other authors [14].

The correlation of Fas-positive cells with other inflammatory markers was of interest. Previously, we observed an elevated proportion of NK cells and NKT-like cells in OSAS patients. NKT cells form a complex population which was found to be an important link in immune regulation [15, 16]. In our study, these two populations show correlation with Fas-positive cells. Fas-positive cells also correlated with HLA-DR+ T cells, which are early-activated lymphocytes. This observation can indicate high turnover of T cells as these activated lymphocytes are prone to activation-induced cell death [17]. Previously, we confirmed the significance of adiponectin in the inflammatory process in OSAS as well as its complications. Adiponectin concentration to BMI ratio (A/BMI) correlated with the OSA complication score: the lower A/BMI index, the higher the risk of cardiovascular and metabolic complications of OSAS [3]. This study presents an important finding: Fas-positive cells and the concentration of soluble Fas were accompanied by a low value of A/BMI ratio. It shows the direction of immune response in OSAS, independent of metabolic disorders. We found a negative correlation of Fas-positive lymphocytes with neutrophils and platelets. The data concerning the value of neutrophil/lymphocyte and platelet/lymphocyte ratio in OSA in prediction of cardiovascular complications are conflicting and seem to be speculative [18–20]. Dyugovskaya et al. described prolonged survival of neutrophils in hypoxic conditions by inhibition of apoptosis [21]. Taken together, the associations of hypoxia and apoptotic pathways in OSAS need careful investigation.

The major weakness of our study was that we did not perform functional analysis of Fas-positive lymphocytes. In conclusion, we present for the first time the association of Fas-positive lymphocytes with the systemic inflammation of OSAS.

## References

[1] de Lima FF, Mazzotti DR, Tufik S, Bittencourt L (2016). The role inflammatory response genes in obstructive sleep apnea syndrome: a review. Sleep Breath.

[2] Wang J, Yu W, Gao M, Zhang F, Gu C, Yu Y, Wei Y (2015) Impact of obstructive sleep apnea syndrome on endothelial function, arterial stiffening, and serum inflammatory markers: an updated meta-analysis and metaregression of 18 studies. J Am Heart Assoc 4(11). 10.1161/JAHA.115.002454 10.1161/JAHA.115.002454 PMC4845236 26567373

[3] Domagala-Kulawik J, Osinska I, Piechuta A, Bielicki P, Skirecki T (2015). T, B, and NKT Cells in Systemic Inflammation in Obstructive Sleep Apnoea. Mediat Inflamm.

[4] Guegan JP and Legembre P (2017) Nonapoptotic functions of Fas/CD95 in the immune response. FEBS J 285(5):809–827. 10.1111/febs.14292 10.1111/febs.14292 29032605

[5] Le Gallo M, Poissonnier A, Blanco P, Legembre P (2017). CD95/Fas, Non-Apoptotic signaling pathways, and kinases. Front Immunol.

[6] Yamada A, Arakaki R, Saito M, Kudo Y, Ishimaru N (2017). Dual role of Fas/FasL-Mediated signal in peripheral immune tolerance. Front Immunol.

[7] The Report of an American Academy of Sleep Medicine Task Force (1999). Sleep-related breathing disorders in adults: recommendations for syndrome definition and measurement techniques in clinical research. Sleep.

[8] Plywaczewski R, Brzecka A, Bielicki P, Czajkowska-Malinowska M, Cofta S, Jonczak L, Radlinski J, Tazbirek M, Wasilewska J (2013). [Sleep related breathing disorders in adults - recommendations of Polish Society of Lung Diseases. Pneumonol Alergol Pol.

[9] Dyugovskaya L, Lavie P, Hirsh M, Lavie L (2005). Activated CD8+ T-lymphocytes in obstructive sleep apnoea. Eur Respir J.

[10] Tan HL, Gozal D, Wang Y, Bandla HP, Bhattacharjee R, Kulkarni R, Kheirandish-Gozal L (2013). Alterations in circulating T-cell lymphocyte populations in children with obstructive sleep apnea. Sleep.

[11] Kyaw T, Peter K, Li Y, Tipping P, Toh BH, Bobik A (2017). Cytotoxic lymphocytes and atherosclerosis: significance, mechanisms and therapeutic challenges. Br Aust J Pharm.

[12] Domagala-Kulawik J, Hoser G, Dabrowska M, Chazan R (2007). Increased proportion of Fas positive CD8+ cells in peripheral blood of patients with COPD. Respir Med.

[13] Hoser G, Wasilewska D, Domagala-Kulawik J (2004). Expression of Fas receptor on peripheral blood lymphocytes from patients with non-small cell lung cancer. Folia Histochem Cytobiol.

[14] Baffi CW, Wood L, Winnica D, Strollo PJ, Gladwin MT, Que LG, Holguin F (2016). Metabolic syndrome and the lung. Chest.

[15] Gaoatswe G, Kent BD, Corrigan MA, Nolan G, Hogan AE, McNicholas WT, O'Shea D (2015). Invariant natural killer T cell deficiency and functional impairment in sleep apnea: links to cancer comorbidity. Sleep.

[16] Hogan AE, Corrigan MA, O'Reilly V, Gaoatswe G, O'Connell J, Doherty DG, Lynch L, O'Shea D (2011). Cigarette smoke alters the invariant natural killer T cell function and may inhibit anti-tumor responses. Clin Immunol.

[17] Krueger A, Fas SC, Baumann S, Krammer PH (2003). The role of CD95 in the regulation of peripheral T-cell apoptosis. Immunol Rev.

[18] Altintas N, Cetinoglu E, Yuceege M, Acet AN, Ursavas A, Firat H, Karadag M (2015). Neutrophil-to-lymphocyte ratio in obstructive sleep apnea; a multi center, retrospective study. Eur Rev Med Pharmacol Sci.

[19] Koseoglu HI, Altunkas F, Kanbay A, Doruk S, Etikan I, Demir O (2015). Platelet-lymphocyte ratio is an independent predictor for cardiovascular disease in obstructive sleep apnea syndrome. J Thromb Thrombolysis.

[20] Koseoglu S, Ozcan KM, Ikinciogullari A, Cetin MA, Yildirim E, Dere H (2015). Relationship between neutrophil to lymphocyte ratio, platelet to lymphocyte ratio and obstructive sleep apnea syndrome. Adv Clin Exp Med.

[21] Dyugovskaya L, Polyakov A, Lavie P, Lavie L (2008). Delayed neutrophil apoptosis in patients with sleep apnea. Am J Respir Crit Care Med.

